# Supplementary material for: Mechanisms governing the pioneering and redistribution capabilities of the non-classical pioneer PU.1
Source: Nat Commun. 2020 Jan 21;11:402. doi: 10.1038/s41467-019-13960-2 (PMC6972792; doi:10.1038/s41467-019-13960-2)
Supplement: Supplementary file 7 — Source data [file 41467_2019_13960_MOESM7_ESM.zip › Source_Data/FigureS2/FigureS2B_MotifScanOutput/KmeansCluster12/homerResults/motif9.similar.html]

motif9

## Information for motif9

T
C
G
A
A
C
G
T
A
C
T
G
C
G
T
A
T
A
G
C
C
G
A
T
G
T
A
C
G
T
C
A
A
G
C
T
G
T
A
C
  
Reverse Opposite:  

C
A
T
G
T
C
G
A
A
C
G
T
C
A
T
G
G
C
T
A
A
T
C
G
A
C
G
T
T
G
A
C
C
G
T
A
A
G
C
T
  

|  |  |
| --- | --- |
| p-value: | 1e-41 |
| log p-value: | -9.444e+01 |
| Information Content per bp: | 1.644 |
| Number of Target Sequences with motif | 143.0 |
| Percentage of Target Sequences with motif | 7.96% |
| Number of Background Sequences with motif | 888.8 |
| Percentage of Background Sequences with motif | 1.93% |
| Average Position of motif in Targets | 103.8 +/- 51.9bp |
| Average Position of motif in Background | 101.6 +/- 60.8bp |
| Strand Bias (log2 ratio + to - strand density) | 0.1 |
| Multiplicity (# of sites on avg that occur together) | 1.07 |
| Motif File: | file (matrix) reverse opposite |

### Similar de novo motifs found

|  |  |  |  |  |  |  |  |
| --- | --- | --- | --- | --- | --- | --- | --- |
| Rank | Match Score | Redundant Motif | P-value | log P-value | % of Targets | % of Background | Motif file |
| 1 | 0.971 | A C T G T C G A C A T G T G C A A C G T A T C G C G T A A T C G C A G T T G A C G C T A A C G T | 1e-40 | -93.440693 | 10.18% | 3.08% | motif file (matrix) |
| 2 | 0.980 | T C G A A C G T A C T G C G T A A T C G A C G T A G T C C G T A A G C T G T A C A C G T | 1e-37 | -86.729079 | 7.57% | 1.90% | motif file (matrix) |
| 3 | 0.955 | A C G T A C T G C G T A A T C G A C G T A G T C C G T A A C G T G T A C | 1e-33 | -77.924319 | 10.41% | 3.64% | motif file (matrix) |
| 4 | 0.967 | A G C T A C T G G T C A T A C G A C G T A T G C C T G A A G C T | 1e-33 | -77.689456 | 11.30% | 4.18% | motif file (matrix) |
| 5 | 0.914 | A C G T A C T G C G T A A G T C A C G T A G T C C G T A | 1e-31 | -71.879731 | 9.52% | 3.31% | motif file (matrix) |
| 6 | 0.824 | C A T G T C G A T G A C T G C A A C G T A T C G T G C A T A G C A C G T A T G C T C G A A T C G A G T C | 1e-20 | -47.766028 | 2.67% | 0.42% | motif file (matrix) |
| 7 | 0.815 | T A C G T G C A A C G T A T C G G T C A A T C G A G C T A G T C C G T A A C G T G T A C G C T A A T G C A G T C | 1e-13 | -29.974963 | 1.17% | 0.11% | motif file (matrix) |
